# Differential Expression of TXNIP Isoforms in the Peripheral Leukocytes of Patients with Acute Myocardial Infarction

**DOI:** 10.1155/2018/9051481

**Published:** 2018-06-21

**Authors:** Yujing Zhang, Peng Zhong, Yingze Xu, Baokui Wang, Tao Zhu, Wendi Zhang, Haihua Wang, Zixiu Wei, Jian Huang

**Affiliations:** ^1^Department of Cardiology, Jining No.1 People's Hospital, Jining 272000, China; ^2^Central Laboratory, Affiliated Hospital of Jining Medical University, Jining 272029, China

## Abstract

**Background:**

Acute myocardial infarction (AMI) is the most serious type of coronary atherosclerotic heart disease (CAD). The pathological changes are characterized by atherosclerosis. Oxidative stress plays an important role in atherosclerosis. Thioredoxin-interacting protein (TXNIP), an endogenous inhibitor and regulator of thioredoxin, could bind thioredoxin to regulate its expression and antioxidant activity negatively. The NCBI data show that there are two isoforms in TXNIP gene, namely, TXNIP1 and TXNIP2. Our previous studies have shown that TXNIP expression levels in patients with unstable angina pectoris (UAP) were increased compared with controls (CTR). However, no upregulation of TXNIP was detected in AMI patients.

**Methods:**

The leucocytes were isolated from peripheral venous blood, and total RNA of the leucocytes was extracted. Then, real-time quantitative PCR was performed.

**Results:**

mRNA levels of TXNIP2 in AMI were significantly increased compared with CTR (*P* < 0.05). However, the expression of TXNIP1 was downregulated in AMI, but the difference was not statistically significant (*P* > 0.05). Logistic regression analysis showed that TXNIP2 mRNA levels were significantly associated with AMI (OR = 2.207, *P* < 0.05).

**Conclusions:**

The expression of TXNIP2, not TXNIP1, is upregulated in leukocytes of AMI patients, indicating that only TXNIP2 in circulating leucocytes may be involved in the pathogenesis of AMI.

## 1. Introduction

Acute myocardial infarction (AMI) is the most serious type of coronary atherosclerotic heart disease (CAD), which seriously endangers human health [1]. Despite the identification of numerous molecular mechanisms, understanding of the pathophysiology of this clinical syndrome remains incomplete. The main risk factors include cigarette smoking, diabetes mellitus, hypertension, and hyperlipidemia [2, 3]. The pathological changes of AMI are characterized by atherosclerosis. Oxidative stress plays an important role in atherosclerosis, which induces vascular-related gene expression, promoting local inflammatory response and cell proliferation. When oxidative stress occurs, vascular walls produce excessive reactive oxygen species (ROS), which causes damage to the structure and function of endothelial cells and enhances the inflammatory response of the vascular wall. ROS participates in various biochemical reactions and is an essential form of energy. But, excessive ROS can lead to disease in pathological conditions. Thioredoxin (TRX) is a multifunctional protein with redox activity, which can act as a neutralizing agent by combining ROS, protecting cells from oxidative stress.

Thioredoxin-interacting protein (TXNIP), a 46 kDa protein originally found in HL-60 cells, also known as Vitamin D3 upregulated protein 1 (VDUP1), is an endogenous inhibitor and regulator of TRX [4–6]. TXNIP could bind TRX to negatively regulate its expression and antioxidant activity [7–9]. And TXNIP negatively regulates the expression of JNK, P38, and VCAM1, increases vascular inflammation, and accelerates the process of atherosclerosis [10]. The inducible nature of TXNIP under several stress conditions, including UV light, *γ* rays, heat shock, and high glucose suggested that TXNIP may play a role in the cellular processes of cell differentiation, apoptosis, immune response, and energy metabolism [11]. Furthermore, it was found that TXNIP overexpression renders the cells more vulnerable to oxidative stress [5, 12, 13]. TXNIP gene expression could be induced by many stress factors, such as heat shock and starvation. On the other hand, hypoxia, nitric oxide (NO), and FOXO1 could inhibit its expression [14]. Recent studies have shown that TXNIP contributes to some of the pathological consequences of myocardial ischemia and infarction through endogenous signals in multiple molecular mechanisms [7, 15, 16].

**Figure 1:**
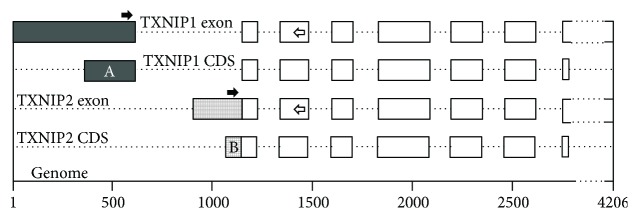
Comparison of difference between TXNIP1 and TXNIP2 coding sequences. NCBI data showed that the TXNIP gene was located on the complementary strand of chromosome 1 (1q21.1, 145992426-145996631, a total of 4206 base pairs), including 8 exons. The exons/CDS with different filling patterns represent the different regions of the two isoforms. The solid arrows represent the position of the forward primers, and the hollow arrows represent the position of the reverse primers. The two forward primers share the same reverse primer. The coding region A contains 250 bases, and the coding region B contains 85 bases. Except for the last base, the nucleotide sequences of the two regions are completely different. That is to say, there are differences in amino acid length and sequence between the N terminal (83 amino acids) of TXNIP1 and the N terminal (28 amino acids) of TXNIP2.

It has been proved that macrophages, lymphocytes, and neutrophils play an important role in atherosclerosis [17, 18]. Because the TXNIP is a ubiquitously expressed protein [11], we speculate that abnormal expression of TXNIP in leukocytes may be associated with coronary heart disease. Furthermore, our previous studies have shown that TXNIP expression levels in patients with unstable angina pectoris (UAP) were significantly increased compared with healthy controls (CTR). However, the situation is different in AMI; there was no significant statistical difference in the expression level of TXNIP between AMI and CTR [19]. The data of the NCBI (National Center for Biotechnology Information) database show that the human TXNIP gene has two isoforms (Figure 1), namely, TXNIP1 (accession: NM_006472.5) and TXNIP2 (accession: NM_001313972.1). We detected the mRNA levels of the two isoforms in peripheral leucocytes of AMI and CTR. And the possible molecular mechanisms were also discussed in the present study.

## 2. Material and Methods

### 2.1. Patients and Controls

From February 2017 to October 2017, a total of 87 AMI patients were recruited from the Department of Cardiology, Jining No.1 People's Hospital, Jining, Shandong, China. All patients were diagnosed with angiography. Ninety age- and sex-matched CTR, without cerebrovascular diseases, were recruited from the Health and Physical Examination Center. All the subjects were coded, and the authors could not identify a single subject. This study was approved by the Human Ethics Committee of Jining No.1 People's Hospital, and informed consents were obtained.

### 2.2. Sample Collection

Peripheral venous blood (5 mL) of the AMI and CTR was collected into EDTA-K2 anticoagulant tube after an overnight fast. The leucocytes were isolated with human leukocyte isolation system (LTS-1078, Haoyang Biological, Tianjin, China), according to the manufacturer's protocol. Total cholesterol (TC), high-density lipoprotein cholesterol (HDL-C), low-density lipoprotein cholesterol (LDL-C), and triglyceride (TG) were determined with an ADVIA® 2400 automated analyzer (Siemens Healthcare Diagnostics, Erlangen, Germany) in the Laboratory of Experimental Medicine.

### 2.3. Real-Time Quantitative PCR (qPCR)

Total RNA of the leucocytes was isolated using TRIzol reagent (15596026, Invitrogen, USA) following the manufacturer's protocol. cDNA was reverse-transcribed using 1.5 *μ*g total RNA as template with RevertAid H Minus First Strand cDNA Synthesis Kit (K1622, Thermo Scientific, USA). qPCR was performed to detect the mRNA levels using SYBR® Premix Ex Taq™ (RR420A, Takara, China) by Real-Time PCR System (Applied Biosystems 7500, USA). Amplification primers (TXNIP1-F: ACG CTT CTT CTG GAA GAC CA, TXNIP2-F: GCA AGC CTA ATG GCT ACT CG, TXNIP-R: AGG GGT ATT GAC ATC CAC CA, *β*-actin-F: GGA CTT CGA GCA AGA GAT GG, *β*-actin-R: AGC ACT GTG TTG GCG TAC AG) were included in the PCR reaction system. The qPCR reaction was set up in a reaction volume of 20 *μ*L containing 10 *μ*L SYBR Premix, 0.5 *μ*L forward primer (10 *μ*M), 0.5 *μ*L reverse primer (10 *μ*M), 2 *μ*L cDNA, and 7 *μ*L nuclease-free water. All samples were performed in triplicate. The PCR operation program was set according to the manufacturer's protocol. The relative expression was calculated with the following equation: relative expression = 2^−ΔΔCt^ [20]. *β*-Actin is the reference gene for normalization.

### 2.4. Statistical Analysis

Data were expressed as means ± standard deviation (X ± SD). Comparisons of means between groups were made by Student's *t*-test and comparisons of prevalence by chi-square test. All statistical analysis were performed with SPSS v22.0. *P* < 0.05 was considered statistically significant.

## 3. Results

### 3.1. Clinical Characteristics

The clinical characteristics are summarized in Table 1. Patients with AMI have a significantly higher prevalence of smoke and a lower HDL-C level (*P* < 0.05). No differences for TG, TC, and LDL-C were detected between CAD and CTR.

### 3.2. TXNIP Isoforms' mRNA Expression Levels

**Figure 2:**
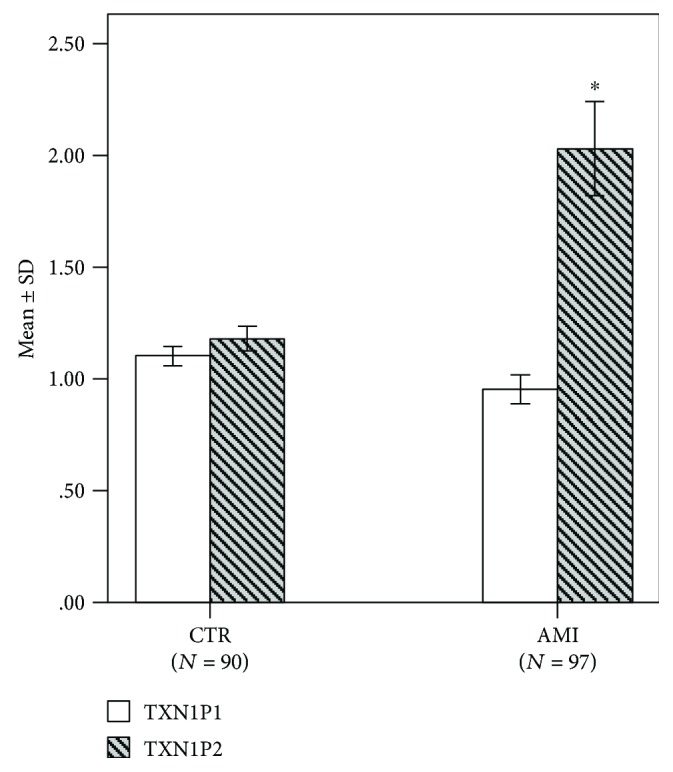
The mRNA expression levels of TXNIP1 and TXNIP2 in AMI and CTR. The calculation method of expression level is as follows: first, we calculate the ΔCt (ΔCt = Ct_TXNIP1/2_ − Ct_*β*-actin_) of all samples and the mean value of ΔCt of the CTR group (ΔCt_CTR_). Then, the ΔΔCt value is calculated as follows: ΔΔCt = ΔCt − ΔCt_CTR_. Finally, the expression of the sample was calculated according to relative expression = 2^−ΔΔCt^. qPCR results showed that the mRNA expression levels of TXNIP1 and TXNIP2 were inconsistent between CTR and AMI. Compared with CTR, the expression of TXNIP1 was downregulated in AMI, but the difference was not statistically significant. The expression of TXNIP2 was increased, and the difference was statistically significant. ^∗^*P* < 0.05.

Our previous studies have shown that TXNIP gene expression levels in patients with UAP were significantly increased compared with CTR but AMI not. So, we detected the mRNA levels of the two variants of TXNIP gene, TXNIP1 and TXNIP2, by qPCR. As shown in Figure 2, mRNA levels of TXNIP2 in AMI patients were significantly increased compared with CTR (*P* < 0.05). However, the mRNA levels of TXNIP1 were completely different from that of TXNIP2. The expression of TXNIP1 was downregulated in AMI, but the difference was not statistically significant (*P* > 0.05).

Further multivariate logistic regression analysis between AMI and TXNIP1 mRNA levels, TXNIP2 mRNA levels, smoking, hypertension, and diabetes was carried out. And the results showed that only TXNIP2 mRNA levels were positively associated with AMI (OR = 2.207, *P* < 0.05). It is important to point out that the mRNA expression levels were redefined in the multivariate logistic regression analysis [21]. The mRNA relative expression “<1.00” was defined as the low expression, and “>1.00” was defined as high expression.

## 4. Discussion

Multiple risk factors lead to AMI, such as smoking, diabetes mellitus, hypertension, hyperlipidemia, inflammatory response, oxidative stress, apoptosis, vascular remodeling, plaque stress, and blood flow shear stress [2, 3, 22]. Our previous studies have shown that the TXNIP gene is upregulated in leukocytes in patients with UAP, but this phenomenon does not exist in AMI patients [19]. The symptoms of UAP are diverse. When the disease just occurs, the symptoms are mild. Most of our subjects had a history of primary hospital visits and began to use certain drugs. Studies have shown that these drugs have an impact on the expression of TRX. So, our previous studies still need to be further validated. Considering the diagnostic criteria and other factors, we only carried out the expression of TXNIP isoforms in AMI.

**Figure 3:**
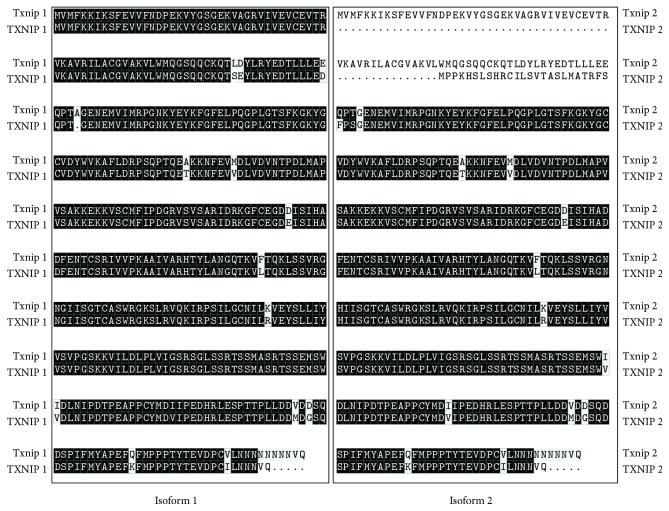
Amino acid sequence similarity of the two isoforms among species. The similarity of amino acid sequence of isoform 1 is 94.46% and that of isoform 2 is 74.75%. Black shades represent the same amino acids, while other shades are different amino acids. Isoform 1 refers to TXNIP1 or Txnip1, and isoform 2 refers to TXNIP2 or Txnip2. All capital letters represent genes of *Homo sapiens*, and the capitalization of the initials is the genes of *Mus musculus*.

TXNIP gene is localized on chromosome 1 (1q21.1), with a total of 8 exons (Figure 1). There are two isoforms, TXNIP1 (391 amino acids and 2983 bp mRNA, NCBI data) and TXNIP2 (336 amino acids and 2614 bp mRNA, NCBI data), which are evolutionally conserved (Figure 3). The difference between the two proteins is mainly at the N end. In this study, we designed primers for differential sequence region of the two isoforms (Figure 1), and then detected their expression levels. The results showed that only TXNIP2 was differentially expressed between AMI and CTR, indicating that TXNIP1 and TXNIP2 may play different roles in the development of AMI. It is also possible that TXNIP1 and TXNIP2 participate in completely different physiological processes.

At present, there are no reports about these two isoforms, and we will continue to explore their functional differences in subsequent studies. Moreover, it is necessary to point out that there are no specific antibodies to these two isoforms in the current reagent companies, and we cannot detect whether there are differences in the protein levels. In theory, the antibody used in our previous studies could detect these two isoforms, but we had detected only one band. In combination with the trend of the expression level of the two variants in this study, we speculate that the expression of the two isoforms may vary greatly; the expression of TXNIP2 is very low and has not been detected in previous studies. In addition, it is necessary to verify whether the two isoforms are completely the same protein after posttranslational modification.

Additionally, there are some defects or limitations in the present study. Firstly, the sample size of this study was relatively small, which could lead to some statistical errors. Secondly, on the choice of the control group, the control population was not validated by angiography, which resulted in asymptomatic coronary heart disease likely to be included in CTR. Thirdly, this study lacks protein level detection, which reduces reliability to some extent. We will complement this study in subsequent studies. Finally, compared to AMI patients, all CTR are negative for risk factors, including smoke, diabetes, and hypertension. Perhaps, the TXNIP2 differences between AMI and CTR are due to risk factors and/or AMI. In our previous study [19], these factors were basically the same, and the difference in the expression level of the TXNIP gene was very close to the results of the TXNIP1 in this study. Therefore, we think that the effects of these risk factors can be ignored, but it still needs further verification.

In conclusion, we found that the mRNA level of TXNIP2, but not TXNIP1, was significantly upregulated in AMI. Further analysis will be continued to verify its role in AMI. These data would shed light on our understandings of the AMI pathogenesis and development of better diagnostic markers.

## 5. Conclusions

The expression of TXNIP2, not TXNIP1, increased in peripheral blood leukocytes of AMI patients, indicating that only TXNIP2 in circulating leucocytes may be involved in the pathogenesis of AMI.

## Figures and Tables

**Table 1:** Clinical characteristics of the normal control and patients.

Item	CTR	AMI
Gender		
Female	36	33
Male	54	54
Smoke		
No	90	50
Yes	0	37
Diabetes		
No	90	63
Yes	0	24
Hypertension		
No	90	61
Yes	0	26
Age	58.08 ± 7.18	60.55 ± 11.23
TG	1.88 ± 0.34	1.38 ± 0.28
TC	5.31 ± 0.20	4.58 ± 0.26
HDL-C	1.47 ± 0.07	1.06 ± 0.06
LDL-C	3.03 ± 0.16	2.63 ± 0.20

TG: triglyceride; TC: total cholesterol; HDL-C: high-density lipoprotein cholesterol; LDL-C: low-density lipoprotein cholesterol. The levels of serum TG, TC, HDL-C, and LDL-C in patients with CTR and AMI were statistically different (*P* < 0.05).

## Data Availability

All data arising from this study are contained within the article.
